# Universal adhesives and dual-cured core buildup composite material: adhesive properties

**DOI:** 10.1590/1678-7757-2020-0121

**Published:** 2020-11-30

**Authors:** Pâmela Malaquias, Mario Felipe Gutiérrez, Elisama Sutil, Thalita de Paris Matos, Taise Alessandra Hanzen, Alessandra Reis, Jorge Perdigão, Alessandro Dourado Loguercio

**Affiliations:** 1 Universidade Estadual de Ponta Grossa Departamento de Odontologia Ponta GrossaPR Brasil Universidade Estadual de Ponta Grossa, Departamento de Odontologia, Ponta Grossa, PR, Brasil.; 2 Universidad de los Andes Facultad de Odontogia Chile Universidad de los Andes, Facultad de Odontologia, Chile. Universidas de Chile, Facultad de odontogia, Instituto de Investigación en Ciencias Odontológica, Chile.; Universidas de Chile Facultad de odontogia Instituto de Investigación en Ciencias Odontológica Chile; 3 University of Minnesota Division of Operative Dentistry Department of Restorative Sciences MinneapolisMN USA University of Minnesota, Division of Operative Dentistry, Department of Restorative Sciences, Minnesota, Minneapolis, MN, USA.

**Keywords:** Self-Curing of Dental Resins, Adhesive, Shear Strength, Dental Leakage

## Abstract

Dual-cured buildup composites and simplified light-cured adhesive systems are mixed with a chemical activator to prevent the incompatibility between them.

**Objective:**

To evaluate microshear bond strength (μSBS) and nanoleakage (NL) of three universal adhesives used under buildup composites using different curing modes, at baseline and after 6-months (6m).

**Methodology:**

Dentin specimens of 55 molars were assigned to: Clearfil Universal Bond[CFU], Prime&Bond Elect[PBE] and One Coat 7 Universal[OCU]. All-Bond Universal[ABU] and Adper Scotchbond Multi-Purpose[SMP] were used as controls. CFU, PBE, and OCU were: light-cured [LC], dual-cured using a self-curing activator [DC], and self-cured, using a self-curing activator and waiting for 20 min [SC]. Upon the application of the adhesive, transparent matrices were filled with a dual-cured buildup composite and light cured, then tested in mSBS. For NL, the specimens were submersed in ammoniacal silver nitrate and sectioned to observe under the SEM. Three-way ANOVA and Tukey's test were applied (α=0.05).

**Results:**

OCU/LC-PBE/LC resulted in higher mean μSBS than ABU/LC. For SMP/DC higher mean μSBS were obtained than for both CFU/DC and OCU/DC (baseline). No universal adhesive was significantly affected by curing mode or storage time. CFU, PBE, and OCU did not undergo significant changes in any curing mode (p>0.05). NL (baseline) PBE/LC resulted in higher %NL compared to ABU/LC. SMP/DC resulted in higher %NL than CFU/DC-OCU/DC. CFU/LC/DC resulted in lower %NL than CFU/SC. PBE/SC resulted in lower %NL than PBE/DC. OCU/LC/SC showed lower %NL than OCU/DC. OCU showed significant lower %NL than CFU and PBE. All CFU groups, as well as OCU/SC, resulted in increased %NL at 6m when compared with baseline.

**Conclusion:**

For universal adhesives used in etch-and-rinse mode, self-cured activator and different curing modes did not influence μSBS. However, some interactions were observed for NL, but this influence was material-specific.

## Introduction

The development of dental materials with increased strength and possibility to reestablish the ideal anatomy of fractured or caries-compromised teeth, especially endodontically treated teeth, represents a significant progress in restorative dentistry. Among available materials, core buildup resin composites associated with adhesive systems have become popular and often used in clinical dental practice.^1^ However, post and core restorations still have a significant clinical failure rate.^2^ In the case of core buildup resin composite materials the failure occurs at the adhesive interface,^3^ mainly when the core buildup resin composite is applied in self-cured (SC) or dual-cured (DC) mode.^4^

When SC and DC core buildup resin composites are associated with simplified adhesives (2-step etch-and-rinse or 1-step self-etch), residual uncured acidic monomers from the oxygen-inhibited layer of the cured adhesives remain in direct contact with the resin composite material.^4^ This reaction results from the contact between the simplified adhesive and the basic catalytic components (aromatic tertiary amines) of chemically-cured composites,^4^^–^^7^ leading to a low rate of polymerization^8^ and possibly affecting bond strength of simplified adhesive systems.^6^^,^^9^ Another potential problem is the creation of a hypertonic environment that draws fluid osmotically from the bonded hydrated dentin through the permeable adhesive layer.^10^ The fluid that migrates to the resin composite-adhesive interface is trapped by the overlying hydrophobic composite as water blisters, ^7^ which act as stress raisers that result in mechanical disruption between the adhesive and the resin composite material. The adverse acid-base reaction^8^ and adhesive permeability^11^ may contribute to the incompatibility between simplified adhesives and both SC and DC core composite materials.

Light-cured adhesive systems are mixed with a self-curing activator aiming to prevent the incompatibility between simplified light-cure adhesives and SC or DC core buildup composites, thus ensuring an adequate polymerization in the deepest areas where the light irradiation may be severely weakened.^12^ However, some studies have shown that the potential incompatibility is not necessarily solved by including a self-curing activator in the bonding procedure.^9^^,^^13^

As universal adhesives are similar to older simplified adhesives, the respective shortcomings may be similar, including incompatibility with SC and DC core buildup resin composites. A recent study evaluated universal adhesives used in self-etch mode associated with core buildup resin composites based on the curing mode. This study showed that use of a self-curing activator influenced bond strength and nanoleakage, but this association varied with different materials.^14^ Mixing a self-curing activator with the adhesive may have reversed the deactivation of aromatic tertiary amine initiator by remaining acidic monomers in the oxygen-inhibited layer of adhesive systems with low pH,^4^ improving chemical compatibility between specific universal adhesives and the core buildup composite material, resulting in higher bond strength.

Most literature on the incompatibility between adhesives and DC cements is based on the previous generation of simplified self-etch adhesives. Manufacturers have recently introduced simplified adhesives that are less hydrophilic (i.e., more hydrophobic) and less permeable to water.^15^ The addition of 10-methacryloyloxydecyl dihydrogen phosphate (or 10-MDP) lends hydrophobicity to universal adhesives, making them more hydrophobic than their predecessors. This change is a result of 10-MDP being very hydrophobic owing to its long carbon chain.^16^

A recent study^14^ assessed microshear bond strength and nanoleakage of universal adhesives used in self-etch mode associated with dual-cure core buildup resin composites. The inclusion of a self-curing activator and distinct polymerization sequences affected microshear bond strength and nanoleakage, but this outcome was material specific. However, there is still a lack of knowledge on the effect of these curing modes on universal adhesives used in the etch-and-rinse strategy.

This study aimed to assess microshear bond strength and nanoleakage of universal adhesives used in etch-and-rinse mode, in association with dual-cure core buildup composite materials as affected by (1) curing mode and (2) water storage. The null hypotheses tested were: (1) dentin bond strength and nanoleakage are not affected when the universal adhesive/core buildup resin composite is bonded using different curing modes, and (2) dentin bond strength and nanoleakage are not affected when the adhesive/core buildup resin composite is stored for six months in water.

## Methodology

### Sample Size Calculation

To estimate the sample size we considered data of means and standard deviations of All-Bond Universal (17.0±3.5 MPa) used in previous study using the same methodology in self-etch mode.^14^ According to www.sealedenvelope.com, the minimal sample size required was 13 dentin specimens in each group to detect a difference of 4 MPa among experimental groups, using a two-sided test with a power of 0.80 and α of 0.05. Two extra dentin specimens were added to compensate for specimens potentially discarded during tooth preparation and restorative procedures.

### Tooth preparation and experimental design

In total, 55 extracted and caries-free human third molars were used. The teeth were collected after obtaining the patients’ informed consent under a protocol approved by the Research Ethics Committee Review Board of the local university. The teeth were disinfected in 0.5% chloramine, stored in distilled water and used within three months after extraction.

**Figure 1:**
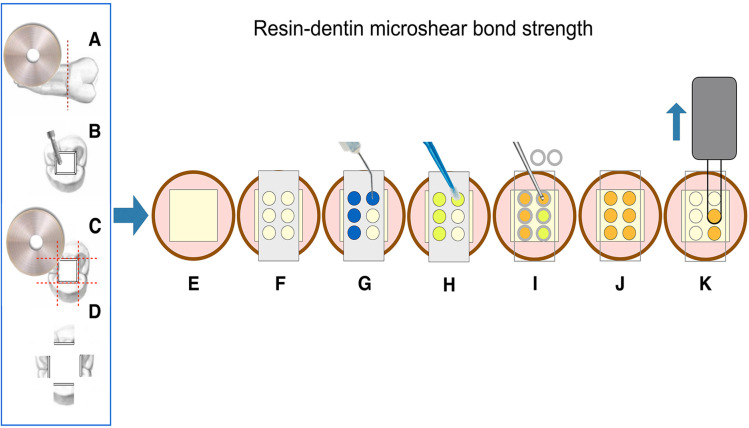
Schematic drawing presenting specimen preparation and testing. (A) The roots of all teeth were sectioned at the cementum–enamel junction. After cavity preparation (B), crowns were sectioned in two perpendicular directions across the long axis of the tooth (C) to produce four dentin specimens (buccal, lingual, and proximals; D). In (E) each dentin specimen was mounted on a PVC ring filled with acrylic resin (displaying the dentin surface on the top of the cylinder); (F) a perforated double-faced adhesive tape was then attached to dentin specimens to delimit the bonding area. After acid etching (G) adhesive application and light curing (H), the Tygon tubes were adapted to the dentin surface (I), and each lumen was filled with core buildup resin composite and polymerized accordingly (J). After each storage time Tygon tubes and adhesive tapes were removed, leaving the bonded core buildup resin composite cylinders on the dentin surface. Each tooth was placed in a jig and assembled in a universal testing machine for microshear bond strength testing using an orthodontic-loop around the core buildup resin composite specimens (K)

The tooth preparation was performed as described by Gutierrez, et al.^14^ (2017). The roots of all teeth were removed by sectioning the enamel-cementum junction (Figure 1A). Then, in each tooth, a Class I cavity (4 mm X 4 mm) was prepared in the occlusal surface with the pulpal floor extending approximately 4 mm into dentin (Figure 1B). The crowns were sectioned longitudinally (Figure 1C) to obtain four 4 mm X 4 mm dentin slabs, each one was obtained from each cavity wall (lingual, buccal, mesial and distal). (Figure 1D). A total of 220 dentin specimens, obtained from 55 teeth, were sanded wet for 60 s each with #600-grit SiC paper and assigned to bond strength (n=165) and nanoleakage (n=55) measurements. The specimens (n=220) were randomly allocated into 11 groups (n=20 specimens per group; 15 for μSBS; 5 for nanoleakage), considering the following independent variables:

*Adhesive (etch-and-rinse)/core buildup resin composite*: Clearfil Universal Bond/Clearfil DC Core Plus [CFU, Kuraray Noritake Dental Inc., Tokyo, Japan]; Prime&Bond Elect/FluoroCore 2+ [PBE, Dentsply Sirona, Milford, DE, USA]; and One Coat 7 Universal/ ParaCore [OCU, Coltene, Altstätten, Switzerland];

*Curing mode*: Three curing modes were used for each of the adhesives CFU, PBE, and OCU: light-cure mode [LC], dual-cure mode [DC] and self-cure mode [SC].

*Two control groups were added*: All-Bond Universal/Core-Flo DC, used as light-cured control adhesive [ABU, Bisco Inc., Schaumburg, IL, USA] and Adper Scotchbond Multi-Purpose/RelyX ARC, used as dual-cured 3-step ER control adhesive [SMP, 3M ESPE, Oral Care, MN, USA];

*Storage time*: Measurements were carried out at 24 hours [baseline] or after 6 months stored in distilled water [6m].

All materials used in this study are similar to those in Gutierrez's study,^14^ except the fact that universal adhesives were applied as self-etch adhesives in the previous work^14^ while in the present one all universal adhesives were applied in the etch-and-rinse strategy.

### Microshear bond strength test (μSBS)

**Figure 2:**
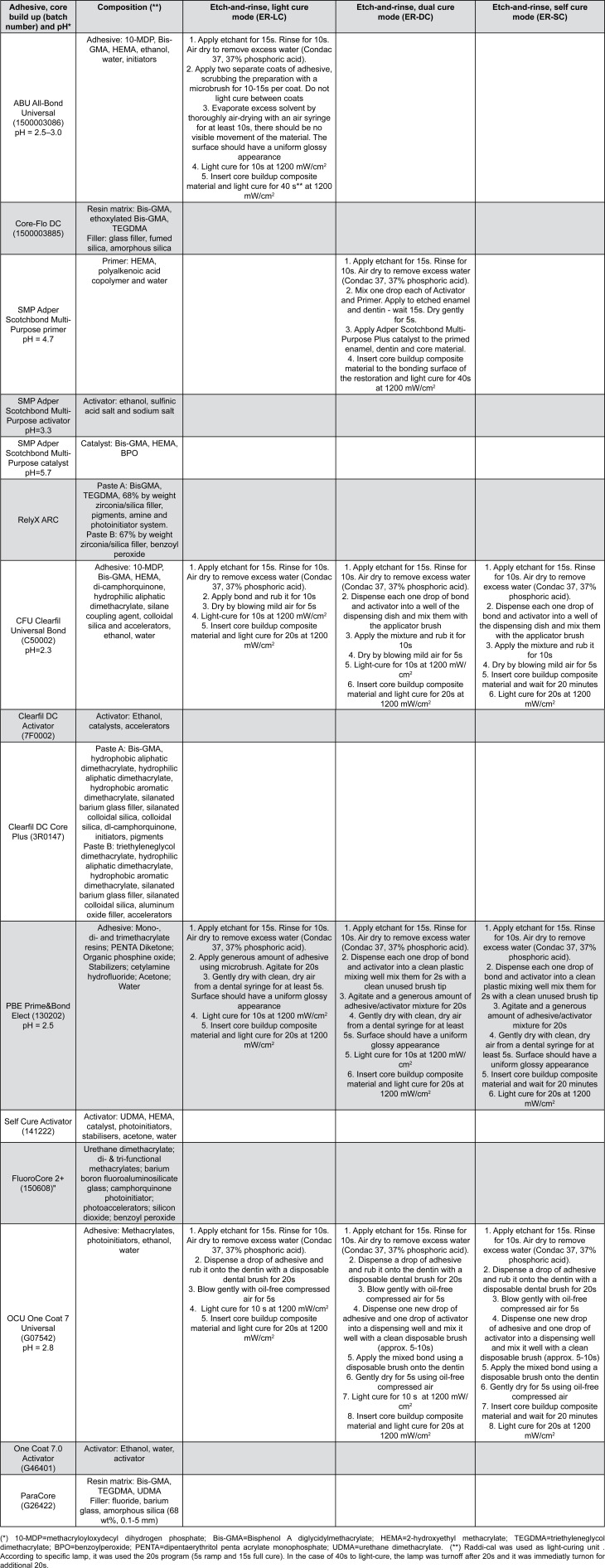
Adhesive and core buildup resin composite system (batch number), pH, composition^*^ and application mode

Acrylic resin (AutoClear, DentBras; Pirassununga, São Paulo, Brazil) was used to fill polyvinyl chloride (PVC) rings. Fifteen random dentin samples in each sub-group were used to evaluate the microshear bond strength (μSBS), after embedding into the acrylic resin extending 3 mm above the PVC ring (Figure 1E). The demarcation of the bonding area was carried out as per Shimaoka, et al.^17^ (2011). Six holes with an internal diameter of 0.8 mm were punched into an acid-resistant double-faced adhesive tape (Adelbras Ind. e Com. Adesivos Ltda, SP, Brazil) with a rubber dam punch (Coltene, Altstätten, Switzerland). This tape was then fixed to the dentin surface (Figure 1F). Prior to adhesive application, all specimens were randomized in block into different groups (www.sealedenvelope.com). A staff member not involved in the research protocol performed this procedure using computer-generated tables. All bonding procedures were carried out by a single operator under a loupe using a magnification of 10X (AmScope, SE305-PZ Binocular Stereo Microscope).^14^ The adhesive systems were then applied in etch-and-rinse mode (Figure 1G) following these group assignments (Figure 2):

All-Bond Universal, light-cure mode (ABU/LC) as a LC control;

Adper Scotchbond Multi-Purpose, dual-cure mode (SMP/DC) as a DC control;

Clearfil Universal Bond, light-cured mode (CFU/LC); dual-cured mode (CFU/DC); and self-cured mode (CFU/SC);

Prime&Bond Elect, light-cured mode (PBE/LC); dual-cured mode (PBE/DC); and self-cured mode (PBE/SC);

OneCoat 7 Universal, light-cured mode (OCU/LC); dual-cured mode (OCU/DC); and self-cured mode (OCU/SC).

The adhesive was applied in three perforations and the solvent was evaporated gently with oil-free compressed air, in the outward direction of the PVC ring to prevent contamination of the other three perforations, and light curing was carried out. The other three perforations were subjected to the same bonding procedure, solvent evaporation in outward direction of the PVC ring to avoid contamination of other perforations where the procedure was already carried out, and light cured. The other three perforations were blocked with an aluminum foil, avoiding a potential increase in polymerization time. This procedure was performed in the same way for adhesive/activator combination, and the complete sequence of Scotchbond Multi-Purpose.^14^

Upon the application of the adhesive (Figure 1H), six transparent cylindrical Tygon tubes (Tygon Medical Tubing Formulations 54-HL, Saint Gobain Performance Plastics, Akron, OH, USA), with an internal diameter identical to that of the perforations (0.8 mm) and a height of 0.5 mm were placed over the tape, ensuring that the respective lumen coincided with the areas uncovered by the perforations. The core buildup resin composite for each adhesive system was carefully packed inside each tube with a stainless #08 K-file. During the restorative procedure, the K-file contacted the Tygon tube wall to fill the inside with the core buildup resin composite and, at the same time, to avoid bubbles. Concomitantly, the Tygon tube was held in position with a precision tweezer (Figure 1I). The core buildup resin composite was photo-polymerized following the respective manufacturer's recommendations (Figure 2) using a LED light-curing unit at 1200 mW/cm^2^ (Radiical, SDI Limited, Bayswater, Victoria, Australia). A radiometer (Demetron L.E.D. Radiometer, Kerr Sybron Dental Specialties, Middleton, WI, USA) was used to verify the light intensity every five specimens. All procedures were carried out under magnification loupes.^14^

The specimens were stored in distilled water at 37°C for the first 24 h. After that, the Tygon tubes and the double-faced adhesive tape were carefully removed with a blade, exposing the composite buildup cylinders (Figure 1J). Each specimen was examined under a stereomicroscope at 10X magnification. If there was evidence of porosities or gaps at the interface, the bonded cylinder was discarded. The composite buildup cylinders from the same dentin specimen were randomly divided, then assigned to test after 24 h [baseline] or after 6 months [6m] in distilled water at 37°C (Figure 1K). The pH of the storage solution was monitored monthly without changing the solution.^14^

The samples were positioned into a testing jig (Odeme Biotechnology, Joaçaba, SC, Brazil), and tested immediately using a universal testing machine (Kratos IKCL 3-USB, Kratos Equipamentos Industriais Ltda, Cotia, SP, Brazil) (Figure 1). After the samples were stabilized onto the testing machine, a thin orthodontic wire with a diameter of 0.2 mm was looped around the base of each composite buildup cylinder. The wire was in contact with the composite buildup sample in half of the external circumference. The setup was kept aligned (interface between composite buildup and dentin, the wire loop, and the center of the load cell) to guarantee the adequate orientation of shear stresses. The crosshead speed was 1 mm/min until failure. The μSBS values were calculated (MPa) by dividing the failure load by the surface area (mm^2^). The failure mode was classified as previously described by Gutierréz, et al.^14^ (2017) as follows: adhesive ([A] failure at the resin–dentin interface), cohesive ([C] failure exclusively within dentin or composite buildup), or mixed ([M] failure at the resin–dentin interface that included cohesive failure of the neighboring substrates). The analysis of the failure mode was carried out with a stereomicroscope (Olympus SZ40, Tokyo, Japan) at a magnification of 100X.

### Nanoleakage evaluation

Five random dentin samples (4 mm X 4 mm) for each group were used for nanoleakage evaluation (NL). All bonding procedures were performed by a single operator (Figure 2). Then, core buildup resin composite was applied on the bonded surfaces in one 2.0 mm-thick increment that was light activated for 40s. A single operator carried out all bonding procedures in a temperature- and moist-controlled environment.^14^

Each sample was divided into two halves by sectioning the enamel-cementum junction in the middle part of the tooth. Each half was randomly assigned to test at 24 h [baseline] or after 6 months [6m] of storage in distilled water at 37°C. The composite buildup-dentin samples were covered with two coatings of nail polish; a rim of 1 mm was left uncoated around the bonded interfaces (Colorama, L'Oréal Brasil, Rio de Janeiro, Brazil). The coated specimens were immersed in 50 wt% ammoniacal silver nitrate solution under darkness for 24 h, rinsed methodically with distilled water, and submersed in a photo developer solution for 8 h under a fluorescent light to reduce silver ions into metallic silver grains within voids along the bonded interface.^14^ The composite buildup-dentin area of the specimens was polished with 1000-, to 4000-grit SiC paper and 1 and 0.25 μm pastes (Buehler Ltd., Lake Bluff, IL, USA). Then, the specimens were cleaned in ultrasonic bath, mounted on aluminum stubs, dried, and sputter-coated with Au (MED 010, Balzers Union, Balzers, Liechtenstein). The interfaces were analyzed under a scanning electron microscope (SEM) in backscattered mode at 12 kV (VEGA 3 TESCAN, Shimadzu, Tokyo, Japan).^14^

The nanoleakage within adhesive and hybrid layer followed the method described by Gutierrez et al.^14^ (2017). Five micrographs were taken for each of the five specimens to standardize image acquisition. The first micrograph was exposed in the center of the composite buildup-dentin specimen. The remaining four micrographs were exposed at 0.3 mm and 0.6 mm to the right and to the left of the first micrograph. A total of five dentin specimens were used for each experimental condition, one dentin specimen per tooth. In total, 25 micrographs were evaluated per group. The micrographs were exposed by a technician who was blinded to the experimental design. The relative percentage of nanoleakage (NL) were measured in all micrographs using a public domain software (Image J), a Java-based image processing software package developed at the National Institutes of Health (NIH).

### Statistical analysis

The experimental unit for μSBS was dentin specimen. For each dentin specimen six Tygon tubes were tested, three after 24h and three after six months. In each storage time, the three Tygon tubes in the same dentin specimen were averaged for statistical purpose. The mean value of μSBS and storage time were obtained for the 15 dentin specimens in each group.

The experimental unit for NL was dentin specimen. For each dentin specimen, after restoration, two halves were obtained, one for each storage time. The five micrographs obtained in the same dentin specimen were averaged for statistical purpose. The mean value of NL for each group and storage time were obtained for the five dentin specimens in each group.

Only specimens with adhesive/mixed failure were averaged for statistical purposes. Specimens with premature and cohesive failures were excluded from data analysis. Data from μSBS and NL were analyzed separately. Prior to evaluation, the data were first analyzed using the Kolmogorov-Smirnov test to assess whether the data followed a normal distribution, as well as Barlett's test for equality of variances to determine if the assumption of equal variances was valid, after observing data normality.

Two statistical analyses were performed: in the first analysis, data were analyzed using three-way ANOVA (adhesive/core buildup resin composites [four levels], curing mode [two levels], and storage time [two levels]). In this first analysis, it was necessary to remove SC groups, mainly because of the absence of a control for these specific groups. In the second analysis, data were analyzed using three-way ANOVA (adhesive/core buildup resin composites [three levels], curing mode [three levels], and storage time [two levels]). In this second analysis, it was not possible to include the control groups (LC and DC). A Tukey's post hoc test at α=0.05 was used for both tests.

## Results

In total, 90 composite buildup cylinders were tested for each group, 45 for each evaluation period. All groups presented more adhesive/mixed failures, ranging between 87% and 100% (Table 1). The preliminary analyses confirmed that there was a normality of the microshear bond strength data distribution and the equality of the variances (data not presented).

**Table 1:** Number of specimens according to fracture mode for all experimental groups (*)

	24-hour water storage (24h)									6-month water storage (6m)								
	LC			DC			SC			LC			DC			SC		
	A	C	M	A	C	M	A	C	M	A	C	M	A	C	M	A	C	M
All-Bond Universal/Core-Flo DC	18	0	27							15	3	27						
Adper Scotchbond Multi-Purpose/RelyX ARC				42	1	2							30	1	14			
Clearfil Universal Bond/Clearfil DC Core Plus	18	1	26	23	1	21	22	2	21	15	4	26	17	6	22	15	3	27
Prime&Bond Elect/Fluorocore 2+	30	0	15	27	1	17	21	2	22	31	2	12	15	2	28	15	5	25
One Coat 7 Universal/ParaCore	25	3	17	27	4	14	16	2	27	20	3	22	18	2	25	26	4	15

(*) A=failure at the resin–dentin interface; C=cohesive (failure exclusively within dentin or resin cement) or; M=mixed (failure at the resin–dentin interface that included cohesive failure of the neighboring substrates).

In the first analysis, the triple cross-product interaction was not significant, as well as the main factor storage time (p>0.05; Table 2). Therefore, only the double cross-product interaction (*adhesive/core buildup resin composites* vs. curing mode) was statistically significant, as well as the main factors *adhesive/core buildup resin composites* vs. curing mode (p<0.01; Table 2). At baseline, the light-curing control group (All-Bond Universal) resulted in similar mean microshear bond strength compared to Clearfil Universal Bond/light-cure (p>0.05; Table 2). Nonetheless, One Coat Universal/light-cure and Prime&Bond Elect/light-cure resulted in statistically significant higher mean microshear bond strength compared with All-Bond Universal (p<0.01; Table 2). In dual-cure mode, the control group Scotchbond Multi-Purpose resulted in statistically significant higher mean microshear bond strength than Clearfil Universal Bond/dual-cure and One Coat Universal/dual-cure (p<0.001; Table 2), but statistically similar to those of Prime&Bond Elect /dual-cure (p>0.05; Table 2). After six months, no statistically significant changes in mean microshear bond strength were measured for either control group (All-Bond Universal and Scotchbond Multi-Purpose) or experimental groups when compared to the respective baseline mean microshear bond strength (p>0.05; Table 2).

**Table 2:** Mean and standard deviation of microshear bond strength (MPa) to dentin for each experimental condition (*,**)

24-hour water storage (24h)			6-month water storage (6m)		
LC	DC	SC	LC	DC	SC
15.6 ± 3.3^C^			15.7 ± 2.9^C^		
	24.9 ± 3.1^A^			22.6 ± 1.6^A,B^	
19.9 ± 1.9^B,C a,b^	15.6 ± 1.1^C b,c^	17.9 ± 3.9^B,C b^	19.3 ± 3.1^B,C a,b^	12.4 ± 3.2^D b,c^	22.8 ± 4.5^A,B a,b^
23.4 ± 2.5^A a^	22.1 ± 3.4^A,B a^	18.4 ± 2.4^B,C a,b^	24.0 ± 3.7^A a^	21.1 ± 4.0^B a,b^	20.2 ± 4.1^B a,b^
21.3 ± 2.7^B a^	21.0 ± 2.9^B a,b^	22.8 ± 2.5^A,B a^	19.2 ± 1.9B,^C a,b^	20.5 ± 2.2^B a,b^	20.2 ± 3.1^B a,b^

(*) Different uppercase letters represent statistically significant differences when LC and DC control groups are compared with respective curing mode of each adhesive/core buildup resin composites (Tukey test, p<0.05).

(**) Different lowercase letters represent statistically significant differences when curing mode of adhesive/core buildup resin composites are compared (no compared with control groups) (Tukey test, p<0.05).

In the second analysis, the triple cross-product interaction was not significant, as well as the double-interactions, and the main factors storage time and curing mode. Therefore, only the main factor *adhesive/core buildup resin composites* was statistically significant (p<0.01; Table 2). No significant changes were observed for Clearfil Universal Bond, Prime&Bond Elect, and One Coat Universal in any curing mode when compared to their respective baseline or 6m mean microshear bond strength (p>0.05; Table 2), as well as, when Clearfil Universal Bond, Prime&Bond Elect, and One Coat Universal were compared in the same curing mode (p>0.05; Table 2). However, when each curing mode was compared for different universal adhesives at baseline, Prime&Bond Elect/dual-cure resulted in statistically significant higher mean microshear bond strength compared to those of Clearfil Universal Bond/dual-cure (p<0.001; Table 3).

**Table 3:** Mean and standard deviation of nanoleakage (%) in dentin for each experimental condition (*,**)

	24-hour water storage (24h)			6-month water storage (6m)		
	LC	DC	SC	LC	DC	SC
All-Bond Universal/Core-Flo DC (control LC)	5.7 ± 3.8^A,B^			5.1 ± 3.7^A,B^		
Adper Scotchbond Multi-Purpose/RelyX ARC (control DC)		22.3 ± 4.1^D^			22.0 ± 4.4^D^	
Clearfil Universal Bond/Clearfil DC Core Plus	5.2 ± 2.7^A,B a,b^	5.2 ± 2.2^A,B a,b^	14.6 ± 5.4^C,D c^	17.8 ± 3.7^C,D c,d^	14.0 ± 5.1^Cc^	20.5 ± 7.4^D d^
Prime&Bond Elect/Fluorocore 2+	14.5 ± 6.7^C,D c^	17.9 ± 3.5^D c^	10.8 ± 4.7^C b^	22.8 ± 4.2^D c^	19.1 ± 5.6^D c^	14.8 ± 5.2^C,D b,c^
One Coat 7 Universal/ParaCore	0.5 ± 1.9^A a^	6.2 ± 2.1^B b^	2.4 ± 1.1^A a^	3.1 ± 1.5^A a^	4.8 ± 3.4^A,B a,b^	8.1 ± 3.4^B b^

* Different uppercase letters represent statistically significant differences when LC and DC control groups are compared with respective curing mode of each adhesive/core buildup resin composites (Tukey test, p<0.05).

** Different lowercase letters represent statistically significant differences when curing mode of adhesive/core buildup resin composites are compared (no compared with control groups) (Tukey test, p<0.05).

### Nanoleakage evaluation

**Figure 3:**
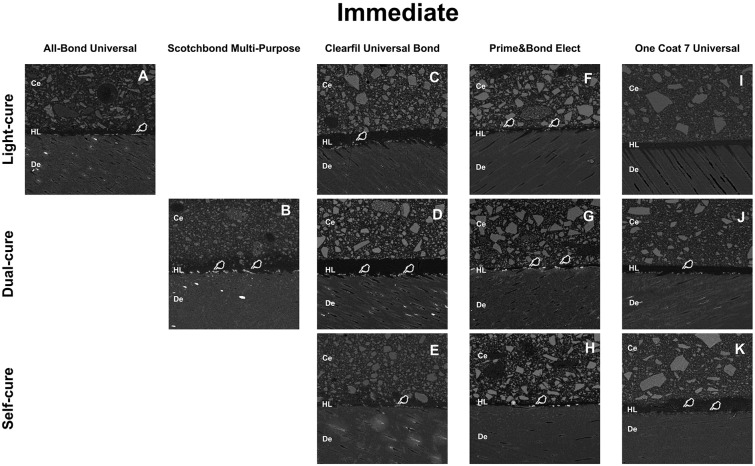
SEM micrographs representations of the resin-dentin interfaces of different experimental groups after 24h-water storage. (Ce=resin cement; De=dentin; HL=hybrid layer)

The preliminary analyses confirmed normality of NL data distribution as well as the variances equality (data not presented). In the first analysis, the triple cross-product interaction was not significant, as well as the main factor storage time (p>0.05; Table 3). Therefore, the double cross-product interaction (*adhesive/core buildup resin composites* vs. curing mode) was statistically significant, as well as the main factors *adhesive/core buildup resin composites* vs. curing mode (p<0.001; Table 3). At baseline, the light-cure control group (All-Bond Universal) resulted in mean NL values similar to those of Clearfil Universal Bond/light-cure and One Coat Universal/light-cure (p>0.05; Table 3). However, Prime&Bond Elect/light-cure resulted in statistically significant higher mean nanoleakage compared to All Bond Universal (p<0.001; Table 3). The dual-cure control group (Scotchbond Multi-Purpose) resulted in similar mean NL when compared with Prime&Bond Elect/dual-cure (p>0.05; Table 3). However, Scotchbond Multi-Purpose showed statistically higher mean NL when compared to Clearfil Universal Bond/dual-cure and to One Coat Universal/dual-cure (p<0.001; Table 3). Figure 3 displays representative SEM micrographs for each group.

In the second analysis, the triple cross-product interaction was significant (p<0.001; Table 2). When each universal adhesive was compared under different curing modes at baseline, Clearfil Universal Bond presented statistically significant lower mean NL in both light-cure and dual-cure modes when compared to Clearfil Universal Bond/self-cure (p<0.001; Table 3). Prime & Bond Elect/self-cure showed statistically significant lower mean NL when compared to Prime & Bond Elect/light-cure and Prime&Bond Elecc/dual-cure (p<0.001; Table 3). One Coat Universal/light-cure and One Coat Universal/self-cure showed statistically significant lower mean NL when compared to One Coat Universal/dual-cure (p<0.001; Table 3). Generally, One Coat Universal resulted in statistically significant lower mean NL when compared with Clearfil Universal Bond and Prime&Bond Elect (p<0.001; Table 3).

**Figure 4:**
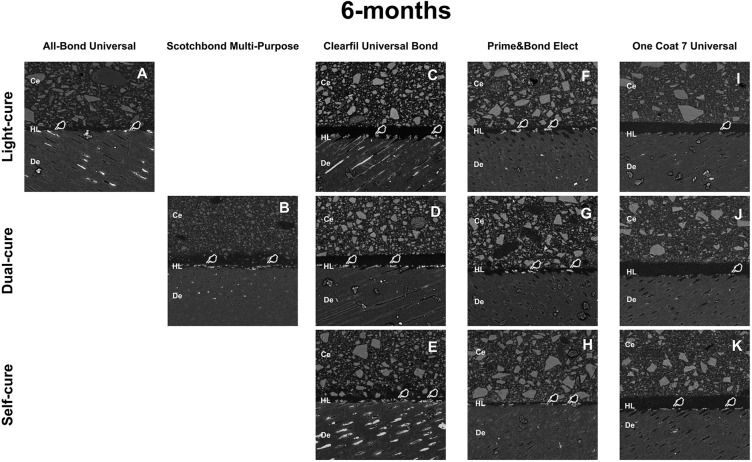
SEM micrographs representation of the resin-dentin interfaces of different experimental groups after 6-month water storage. (Ce=resin cement; De=dentin; HL=hybrid layer)

Clearfil Universal Bond groups presented statistically significant higher mean NL compared to their respective baseline results (p<0.0001; Table 3). A statistically significant increase of mean NL was observed for One Coat Universal only in self-cure mode when compared to the respective baseline results (p<0.0001; Table 3). Although Prime&Bond Elect resulted in the worst (highest) NL (p>0.05; Table 3), there was no increase in NL for Prime&Bond Elect at 6m (p>0.05; Table 3). Figure 4 displays representative SEM micrographs for each group.

## Discussion

In this study, we decided to include two control groups for adequate comparison with universal adhesives that use different curing modes. All-Bond Universal was selected as the control group for the light-cure mode, for All-Bond Universal is a less hydrophilic universal adhesive that contains 10-MDP. Furthermore, All-Bond Universal does not need a self-curing activator with self-cure or dual-cure resin composite materials according to the manufacturer's instructions, due to its relatively high pH (3.2) compared with other universal adhesives.^18^^,^^19^

One Coat Universal and Prime&Bond Elect in light-cure mode resulted in higher bond strengths at baseline when compared to those of All-Bond Universal. In the case of One Coat Universal, its slightly lower amount of HEMA compared to that of All-Bond Universal may have resulted in higher bond strengths in light-cure. Also, the respective manufacturers’ safety data indicate that the concentration of HEMA in One Coat Universal varies between 5-10%, while All-Bond Universal concentration of HEMA varies between 5% and 15%. Considering that HEMA may inhibit interfacial nano-layering of 10-MDP with hydroxyapatite,^20^ the chemical bonding potential of One Coat Universal may have been strengthened compared to other adhesives with higher concentration of HEMA. For Clearfil Universal Bond, its higher amount of HEMA of up to 35%^21^ may have further inhibited the formation of interfacial nano-layering of 10-MDP with hydroxyapatite.

Moreover, the higher amount of solvent in All-Bond Universal (30-60% of ethanol) when compared to One Coat Universal (35-40%) might also be responsible for this significant difference in mean bond strengths. An increased amount of solvent results in more residual solvent being retained in the hybrid layer and adhesive layer,^22^ which prevents the formation of a polymer with high reticulation.^23^ Consequently, bond strengths may be negatively affected. Note that All Bond Universal presented higher mean NL compared to One Coat Universal / light-cure, supporting the hypothesis that HEMA and solvent concentration may affect the adhesive properties.

Prime&Bond Elect/light-cure resulted in higher mean microshear bond strengths in comparison with All Bond Universal and other universal adhesives in light-cure mode. The peer-reviewed literature contains controversial results for Prime&Bond Elect compared to other universal adhesives regarding bond strength.^24^^–^^27^ The presence of acetone in Prime&Bond Elect composition might be responsible for these good results regarding bond strength. In fact, acetone has higher vapor pressure, resulting in rapid solvent evaporation compared to ethanol,^16^ which is present in Clearfil Universal Bond and One Coat Universal, therefore promoting the formation of a polymer with high reticulation that generates higher bond strength as observed in this study.

Unfortunately, the disadvantage of using acetone is that when only one layer of Prime&Bond Elect is applied it may be not enough to achieve a full infiltration of resin monomers into the hybrid layer, causing higher percentage of nanoleakage.^25^ Nanoleakage discloses the location of flaws at the resin-dentin interface that may function as pathways for degradation over time. Silver nitrate ions serve as tracers for nanoleakage, occupying nanometer-sized areas around collagen fibrils that are not enveloped by resin, as resin was unable to infiltrate that area or residual water/solvent was not displaced by the adhesive resin.^28^ Among all universal adhesive evaluated, Prime&Bond Elect contains the highest solvent concentration (below 50%) when compared to that of Clearfil Universal Bond (less than 20%) and One Coat Universal (35-40%). This difference may have been responsible for a greater number of defects (higher %NL) inside the hybrid layer.

In this study, we opted for Scotchbond Multi-Purpose as a dual-cure control, mainly because this material has resulted in higher microshear bond strengths when used in dual-cure mode,^29^ which agrees with our results. The use of chemical co-initiators in Scotchbond Multi-Purpose eliminates the adverse chemical interaction between simplified etch-and-rinse adhesives and self/dual-cured composites.^7^ Another explanation for the good performance of Scotchbond Multi-Purpose regarding bond strength compared to universal adhesives in dual-cure mode may be attributed to the presence of specific polyalkenoic acid copolymer, originally introduced in the composition of the resin-modified glass ionomer Vitrebond (3M Oral Care). Polyalkenoic acid copolymer-containing adhesives bond chemically and spontaneously to hydroxyapatite,^30^ which may explain why an etch-and-rinse adhesive with polyalkenoic acid copolymer showed higher immediate and long-term bond strength than a polyalkenoic acid copolymer-free adhesive.^31^

On the other hand, because polyalkenoic acid copolymer is a compound with high molecular weight, it does not dissolve in the adhesive solution, which may lead to phase separation and formation of resin globules within the polymer.^32^ Furthermore, the dentin collagen network of etched dentin can filter the polyalkenoic acid copolymer out leaving it deposited as a distinct gel on the collagen network surface.^33^ This separation in the polyalkenoic acid copolymer structure results in lower conversion of the adhesive inside the hybrid layer and higher values of nanoleakage.^34^ In agreement with these findings, higher amount of nanoleakage values were also observed in our study for Scotchbond Multi-Purpose when compared to universal adhesives in dual-cure mode.

This study showed that, for all groups a higher number of adhesive/mixed failures, ranging between 87% and 100%, were observed. This indicates that the bond strength method was well executed and these results are in agreement with previously studies when microshear bond strength was applied.^14^^,^^17^ No significant differences were found for all universal adhesives at baseline when the light-cure or dual-cure mode were compared with the self-cure mode, in agreement with previous publications.^14^^,^^24^ Thus, the issue of incompatibility between universal adhesives and dual-cure buildup resin composites may not exist.

It is currently accepted that there is an adverse chemical interaction between the residual unpolymerized oxygen-inhibited layer, which contains residual acidic monomers, and the basic tertiary amine catalysts in the self-cure resin composites/cements.^4^^–^^6^^,^^35^ However, other factors seem to play a role in this incompatibility. It has been observed that simplified etch-and-rinse adhesives behave as permeable membranes after polymerization, mainly because of their greater hydrophilicity.^7^ A rapid water movement due to osmosis may cross the polymerized adhesives leading to physical incompatibility between simplified etch-and-rinse and resin composite materials with chemical initiator.

As indicated in the introduction section, more hydrophobic (i.e., less hydrophilic) simplified adhesives have been developed, which are less water permeable.^15^ For instance, two of the three universal adhesives evaluated in this study in the dual-cure mode (Clearfil Universal Bond and One Coat Universal) contain MDP in their composition, making them less hydrophilic.

Therefore, we theorize that similar mean μSBS measured with different curing modes of all universal adhesives in our study may be caused by their low hydrophilicity, in agreement with Chen and Suh.^36^ In their study,^36^ authors showed that simplified adhesives with the same pH, but with different degrees of hydrophilicity (10-30% of hydroxylethyl methacrylate - HEMA), showed different behavior regarding bonding to dentin. More hydrophobic adhesives (lower % of HEMA) did not result in a decrease of mean bond strength when used with a dual-cure resin cement either light-cured or chemical-cured. According to the description of the respective manufacturers, the universal adhesives evaluated in our study contain lower amount of HEMA in their composition. For One Coat Universal is 5%-10%, whereas for Prime&Bond Elect is less than 20% and for Clearfil Universal Bond is 10-15%, according to the SDS of each manufacturer.

Although no significant changes regarding mean bond strength occurred when different polymerization modes of each universal adhesive were compared, some differences were observed for nanoleakage values. For example, for Prime&Bond Elect the mean NL for the self-cure mode was statistically lower than light-cure mode, both in the immediate and after 6-month of water storage, which may be a result of the buffering characteristics of dentin. In the self-cure mode, the time elapsed between the insertion of the core buildup resin composite and the respective light irradiation (20 minutes) may provide to the acidic adhesive (pH of Prime&Bond Elect = 2.5) ^14^ enough time to interact with dentin. The buffering potential of hydroxyapatite would increase the pH of the Prime&Bond Elect /self-cured activator solution^39^, leading to lower amount of NL inside the hybrid layer. Notably, waiting 20 minutes to light-cure is unrealistic under a clinical point of view. However, in this group, we wanted to simulate a situation when light-curing is not used.

In contrast, this was not observed for One Coat Universal. Such reaction would be less relevant for adhesives with higher pH.^37^ The pH of One Coat Universal (2.8) is slightly higher than the pH of Clearfil Universal Bond (pH = 2.3) and the pH of Prime&Bond Elect (pH = 2.5).^14^^,^^19^ The lower pH of Clearfil Universal Bond along with its higher concentration of HEMA^21^ that precludes nano-layering, may have been responsible for higher mean NL for Clearfil Universal Bond after six months of water storage, as well as Clearfil Universal Bond lower mean bond strengths in self-cure mode when compared with the baseline results. According to the adhesion-decalcification concept^38^ for self-etch adhesives, adhesives with lower pH dissolve more hydroxyapatite crystallites, that precludes the formation of a chemical bond between functional resin monomers and hydroxyapatite in dentin. In spite of the buffering capacity of dentin and the high reactivity of H^+^ being responsible for only allowing a minimal amount of H^+^ to diffuse through dentin, the porous demineralized dentin in the non-demineralized dentin areas of self-etch adhesives may be a result of the accumulation of non-polymerizable hydrolytic adhesive components with low pH in more acidic self-etch adhesives.^19^ These non-polymerizable acidic monomers may even extend the etching effect in the underlying dentin after the formation of the hybrid layer when specimens are stored in water.

It is worth mentioning that, in this study, all universal adhesives were applied in the etch-and-rinse mode. Considering that, 10-MDP establish a very intensive and stable chemical interaction with hydroxyapatite, dissolving the smear layer and the hydroxyapatite on the dentin surface through phosphoric acid etching may reduce chemical interactions mainly in the dentin surface.^38^ Unfortunately, there are significant open questions concerning the interaction between adhesives containing MDP when applied in the etch-and-rinse system. However, a recent study evaluated the effect of phosphoric acid on dentin before the application of a MDP-containing adhesive (commercial) in comparison to a MDP-free adhesive (experimental) from the same manufacturer. The results showed higher bond strength when a MDP-containing adhesive was used, even after phosphoric acid application.^39^ In fact, Hiraishi, et al.^40^ (2013) speculated a certain interaction might occur between exposed collagen fibrils and MDP. On the other hand, it is more plausible the association of methacrylate group with the long carbon spacer group effectively provides hydrophobicity,^38^ and it might contribute to bond durability *in vitro*.

In all groups of this study, the adhesive and core buildup resin composite from the same manufacturer were compared. This approach was followed because, during a luting procedure, the clinician usually applies adhesive and core buildup resin composite from the same manufacturer. Unfortunately, the comparison between adhesive with core build up resin composite from different manufacturers would be difficult to accomplish due to the excessive number of groups. A closer view of the results did not shown any relationship between final results associated to a specific core buildup resin composite used. However, future studies are necessary to confirm the present hypothesis. Summarizing, the first null hypothesis was partially rejected, as the bond strength and nanoleakage values of universal adhesives changed depending on the curing mode used. The second null hypothesis was rejected, as the means of bond strength and nanoleakage of some universal adhesives varied after 6m of water storage.

## Conclusions

For etch-and-rinse universal adhesives, the addition of a self-curing activator and different curing modes did not influence bond strength to dentin. Regarding nanoleakage, some interactions were observed, but this influence was material dependent. On the other hand, the water storage time influenced negatively NL, but in the same way, this influence was adhesive dependent.
